# Potential role of differential medication use in explaining excess risk of cardiovascular events and death associated with chronic kidney disease: A cohort study

**DOI:** 10.1186/1471-2369-12-44

**Published:** 2011-09-14

**Authors:** Nisha Bansal, Chi-yuan Hsu, Malini Chandra, Carlos Iribarren, Stephen P Fortmann, Mark A Hlatky, Alan S Go

**Affiliations:** 1 Department of Medicine, University of California, San Francisco, San Francisco, CA, USA; 2 Division of Research, Kaiser Permanente of Northern California, Oakland, CA, USA; 3 Department of Medicine, Stanford University, Palo Alto, CA, USA

## Abstract

**Background:**

Patients with chronic kidney disease (CKD) are less likely to receive cardiovascular medications. It is unclear whether differential cardiovascular drug use explains, in part, the excess risk of cardiovascular events and death in patients with CKD and coronary heart disease (CHD).

**Methods:**

The ADVANCE Study enrolled patients with new onset CHD (2001-2003) who did (N = 159) or did not have (N = 1088) CKD at entry. The MDRD equation was used to estimate glomerular filtration rate (eGFR) using calibrated serum creatinine measurements. Patient characteristics, medication use, cardiovascular events and death were ascertained from self-report and health plan electronic databases through December 2008.

**Results:**

Post-CHD event ACE inhibitor use was lower (medication possession ratio 0.50 vs. 0.58, P = 0.03) and calcium channel blocker use higher (0.47 vs. 0.38, P = 0.06) in CKD vs. non-CKD patients, respectively. Incidence of cardiovascular events and death was higher in CKD vs. non-CKD patients (13.9 vs. 11.5 per 100 person-years, P < 0.001, respectively). After adjustment for patient characteristics, the rate of cardiovascular events and death was increased for eGFR 45-59 ml/min/1.73 m^2 ^(hazard ratio [HR] 1.47, 95% CI: 1.10 to 2.02) and eGFR < 45 ml/min/1.73 m^2 ^(HR 1.58, 95% CI: 1.00 to 2.50). After further adjustment for statins, β-blocker, calcium channel blocker, ACE inhibitor/ARB use, the association was no longer significant for eGFR 45-59 ml/min/1.73 m^2 ^(HR 0.82, 95% CI: 0.25 to 2.66) or for eGFR < 45 ml/min/1.73 m^2 ^(HR 1.19, 95% CI: 0.25 to 5.58).

**Conclusions:**

In adults with CHD, differential use of cardiovascular medications may contribute to the higher risk of cardiovascular events and death in patients with CKD.

## Background

Cardiovascular diseases remain the leading cause of death in patients with chronic kidney disease (CKD) [1-5]. Potential modification of cardiovascular disease risk through pharmacological interventions is a critical component of current care and prevention in patients with known coronary heart disease (CHD) or at risk for CHD.

Observational studies have shown that despite being at high risk for CHD, many patients with CKD or end-stage renal disease (ESRD) are less likely to receive cardiovascular medications [6-8]. One study found that among the 35.5% of CKD patients not taking an angiotensin converting enzyme (ACE) inhibitor, over half did not have clear contraindications to ACE inhibitor use [9]. In certain studies, CKD or ESRD patients hospitalized with acute myocardial infarction were less likely to receive immediate evidence-based therapies such as aspirin, β-blockers, ACE inhibitors, angiotensin receptor blockers (ARBs) or lipid lowering agents [10-17].

Although previous studies have established that patients with CKD have substantial cardiovascular disease burden [18-20] and are frequently under-prescribed beneficial cardiovascular medications, the potential impact of cardiovascular medications exposure on recurrent cardiovascular events in those with CKD and known CHD is not known. Underuseof cardioprotective medications may be one explanation for the excess cardiovascular morbidity in this at-risk patient population [21]. To address this, we examined whether differential cardiovascular drug use, specifically statins, β-blockers, calcium channel blockers, ACE inhibitors and ARBs, helps to explain, at least in part, the excess risk of recurrent cardiovascular disease events associated with CKD in a large prospective cohort of patients with known CHD followed longitudinally.

## Methods

### Study sample and study design

The source population included adults (age ≥ 20 years) who received medical care within Kaiser Permanente of Northern California, a large integrated healthcare delivery system providing comprehensive care to more than 30% of insured adults in the San Francisco and greater Bay area. Previous studies have shown that the membership is representative of the local surrounding and statewide insured adult population, with the exception of slightly lower proportions of persons at the extremes of age and income level [22]. Adult members of Kaiser between the ages of 45 to 75 years with no prior history of CHD who first presented with new symptoms of CHD (either stable exertional angina or acute myocardial infarction) between October 28, 2001 and December 31, 2003 were enrolled into the Atherosclerotic Disease, VAscular functioN, and genetiC Epidemiology (ADVANCE) Study [23]. Recruitment of study participants, exlusion and inclusion criteria into the ADVANCE study has been described in detail previously [23]. Institutional review boards of the collaborating institutions at Kaiser Permanente Northern California and Stanford University approved the study, and written informed consent was obtained from all study participants.

To identify cases of incident acute myocardial infarction, we identified men aged 45 to 75 years old and women aged 55 to75 years old who had acute myocardial infarction as their first presentation of clinical coronary disease between October 28, 2001 through December 31, 2003 by weekly searches of automated laboratory for elevated cardiac enzymes and hospital discharge databases for primary discharge diagnosis of myocardial infarction (ICD-9-CM code 410). Primary care physicians and patients were screened by telephone interview to confirm the absence of prior diagnosed coronary heart disease, coronary revascularization, or ischemic symptoms more than 14 days before admission for acute myocardial infarction. In addition, electrocardiograms were systematically reviewed to exclude any patients with a prior silent Q-wave myocardial infarction. To identify cases of incident stable exertional angina, we identified men and women aged 45 to 75 years old who had stable exertional angina as their first presentation of clinical coronary disease during this same time period by performing weekly searches of automated ambulatory visit databases for new outpatient diagnoses of angina pectoris (ICD-9-CM 413.x). Primary treating physicians of potential participants were contacted to confirm the occurrence of new onset, stable exertional angina. Patients were similarly screened by telephone interview to confirm the absence of prior coronary heart disease and coronary revascularization. In addition, patients had to report evidence of stable chest pain or chest pressure reproduced by the same level of physical exertion, lasting more than one minute and less than 15 minutes, and responded to rest or nitroglycerin.

We conducted a parallel cohort study of participants of ADVANCE followed from the index date of initial CHD event until December 31, 2008 to assess differential patterns of medication use and rates of recurrent cardiovascular events after an incident CHD event. The exposed group included those with pre-existing chronic kidney disease (CKD), defined as estimated glomerular filtration rate (eGFR) < 60 ml/min/1.73 m^2^. The unexposed, control group included participants of ADVANCE without pre-existing CKD, and were also followed longitudinally for the same time period.

### Measurement of kidney function

Kidney function was assessed using estimated glomerular filtration rate calculated from the latest serum creatinine measurement prior to the index date. Estimated glomerular filtration rate (eGFR) was estimated using the four-variable abbreviated Modification of Diet in Renal Disease (MDRD) Study equation [24] based on the latest outpatient serum creatinine test result found in health plan laboratory databases prior to the index date [25]. Based on prior work and recent guidelines published by the Kidney Disease: Improving Global Outcomes (KDIGO) Committee, we staged kidney function as (in units of ml/min/1.73 m^2^): 90 to 130, 60 to 89, 45-59, and less than 45 [18,26]. As previously described, serum creatinine values were calibrated to the core laboratory used to generate the MDRD estimating equation [18].

### Measurement of medication use

Medication exposure was obtained from health system pharmacy databases. Medication exposure in days was measured from enrollment until death, disenrollment or the end of the study period December 31, 2008. The medication possession ratio (MPR), the number of days of exposure to medication divided by the number of days of follow-up from enrollment date, was calculated for each class of medications (statins, β-blockers, calcium channel blockers, ACE inhibitors, ARBs) for both the study cohort and controls, which has been validated in previous studies as a summary measure of possible medication adherence [27-29]. If a patient was not prescribed a particular medication class, the MPR was assigned a value of zero.

### Covariates

Data were collected on demographic characteristics, lifestyle factors, cardiovascular and other medical history, family history of coronary heart disease, systolic and diastolic blood pressure, and body mass index [30]. Age at index date was based on self-report and confirmed in health plan databases. Patients also provided self-reported information on gender, race/ethnicity, marital status, employment status, annual household income, parental and sibling history of coronary heart disease, history of prior stroke, peripheral arterial disease, diabetes mellitus, hypertension, smoking status at index date (current, former, or never), alcohol use and intensity of leisure-time activity during the 12 months prior to study visit date. The most recent outpatient systolic and diastolic blood pressure values before index date were obtained from ambulatory visit databases, which have been shown to reliably reflect chronic blood pressure levels in our database [31]. Body mass index (kg/m^2^) was measured at the study visit using standard procedures.

### Outcomes

The primary outcome was the occurrence of a recurrent cardiovascular disease event (i.e., hospitalization for acute myocardial infarction, angina, coronary artery bypass graft surgery, percutaneous coronary intervention, peripheral arterial disease, revascularization or stroke) identified from hospital discharge diagnosis and billing claims databases using validated *International Classification of Diseases, Ninth Edition *(ICD-9) codes as well as deaths identified from health plan administrative databases, Social Security Administration vital status files, and California state death certificate registry during follow-up [18]. Case ascertainment was extremely reliable as the majority of events were captured within the health delivery system. The number and type of events were followed for the exposed group with CKD and the unexposed control group without CKD from enrollment until death, disenrollment or the end of the study period on December 31, 2008 which was the latest date with complete death information at the time of analysis. For patients who had more than one event during the follow-up period, the first outcome of its kind was included in the results (e.g. the first occurrence of acute myocardial infarction). Incidence rates were reported in rates per 100 person-years for each event type.

### Statistical approach

All analyses were performed using SAS statistical software version 9.1 (Cary, N.C.). Differences between patients with CKD and without CKD were compared using Student's t test for continuous variables and chi-squared test for categorical variables. We performed Cox proportional hazards regression to examine the association between level of pre-event kidney function and the risk of recurrent cardiovascular disease event, with eGFR ≥ 60 ml/min/1.73 m^2 ^as the referent level of kidney function. Variables included in models were based on variables that were significantly different between study population and controls on bivariate analyses or have previously been shown to be associated either with kidney function or cardiovascular disease. Patient demographic characteristics, lifestyle factors and comorbid conditions were added to regression models. MPRs for statins, β-blockers, calcium channel blockers, ACE inhibitors and ARBs were included in the final model to evaluate whether post-event medication exposure mediated the risk of recurrent cardiovascular events or death in those with CKD.

## Results

The study included 159 patients with CKD and 1088 patients without CKD before their incident CHD diagnosis. Patients with CKD were more likely than those without CKD to be older and women (Table 1). Those with CKD were less likely to engage in moderate or heavy activities compared with the non-CKD group. Mean eGFR was 49.7 ± 9.8 ml/min/1.73 m^2 ^for those with CKD and 85.0 ± 17.7 ml/min/1.73 m^2 ^for those without CKD.

**Table 1:** Baseline characteristics of ADVANCE subjects with incident coronary heart disease at enrollment by CKD status*

Characteristic	CKD (N = 159)	Non-CKD (N = 1088)	P Value
Mean (SD) Age, year	66.8 (7.9)	61.7 (8.3)	< 0.001

Women, %	42.8	25	< 0.001

African American	6.3	3.9	

Mean (SD) prior estimated glomerular filtration rate (ml/min/1.73 m^2^)	49.7 (9.8)	85 (17.7)	< 0.001

Married/Domestic Partner	66.7	75.1	< 0.001

Current cigarette smoking, %	7.6	9.0	0.3

Alcohol use in prior 12 months, %	67.3	69.8	0.5

Leisure-time Activity in past 12 months, %			0.02

Minimal	42.8	31.0	

Light	12.6	18.9	

Moderate	31.5	35.8	

Heavy	13.2	14.3	

Medical History, %			

Stroke/transient ischemic attack	17.0	9.4	< 0.001

Peripheral arterial disease	13.8	9.4	0.08

Diabetes mellitus	34.6	26.9	0.04

Diagnosed hypertension	90.6	79.5	< 0.001

Parental History of Coronary Disease, %	45.9	51.4	0.2

Sibling History of Coronary Disease, %	25.8	22.9	0.4

Mean systolic blood pressure (mm Hg)	132.0	122.0	< 0.001

Systolic (mm Hg), %			< 0.001

≤ 120	31.7	52.2	

121-129	19.0	18.7	

130-139	16.5	13.1	

140-159	19.0	12.3	

160-179	11.4	2.8	

> 180	2.5	1.0	

Mean diastolic blood pressure (mm Hg)	73.0	71.1	0.02

Diastolic (mmHg), %			< 0.001

≤ 80	81.5	84.7	

81-84	6.4	8.5	

85-89	4.5	4.1	

90-99	4.5	2.6	

100-109	1.9	0.2	

≥ 110	1.3	0	

Mean (SD) total cholesterol	215.2 (41.4)	214.9 (41.8)	0.9

Mean (SD) LDL cholesterol	126.9 (32.8)	131.7 (35.7)	0.2

Mean (SD) HDL cholesterol	47.2 (14.4)	44.5 (12.2)	0.02

Mean (SD) triglyceride level	201.2 (139.0)	200.4 (130.2)	0.9

Mean (SD) body mass index (kg/m^2^)	29.6 (5.1)	29.1 (5.4)	0.4

*CKD indicates chronic kidney disease

Patients with pre-existing CKD were more likely than those without CKD to have a history of stroke, diabetes mellitus and hypertension. Mean body mass index was similar in both groups (Table 1). CKD patients were more likely to have elevated blood pressure compared with patients who did not have CKD (Table 1).

At baseline, only 1.3% of patients with CKD were at the current guideline-based target blood pressure (defined as < 130/80 mm Hg for those with CKD or diabetes mellitus and < 140/90 mm Hg for all others) compared with 1.8% of those without CKD. At the end of the follow-up period, 30.2% of those with CKD were at goal blood pressure compared with 19.2% in non-CKD patients.

At baseline only 0.6% of those with CKD had an LDL cholesterol level < 70 mg/dL compared with 1.9% of those without CKD. At the end of the follow-up period, 30.2% of those with CKD achieved an LDL < 70 mg/dL compared with 27.0% of those without CKD.

At the end of the follow-up period, those with CKD achieved a greater reduction in systolic and diastolic blood pressure (Table 2). Both groups were successful in achieving an improvement of their lipid profiles over time, with no significant difference between the CKD and non-CKD group (Table 2).

**Table 2:** Change in cardiovascular risk factors from baseline to end of follow-up period by CKD status*

Characteristic	CKD (N = 159)	Non-CKD (N = 1088)	P Value
**Systolic blood pressure, mean (SD) (mm Hg)**	-2.4 (22.9)	3.2 (21.2)	0.01

**Diastolic blood pressure, mean(SD) (mm Hg)**	-2.9 (13.2)	0.36 (11.6)	< 0.001

**Total cholesterol, mean (SD) (mg/dL)**	-55.9 (50.5)	-54.7 (43.8)	0.7

**LDL cholesterol, mean (SD) (mg/dL)**	-44.3 (39.9)	-46.3 (37.1)	0.6

**HDL cholesterol, mean (SD) (mg/dL)**	0.22 (11.7)	0.94 (9.9)	0.5

**Triglycerides, mean (SD) (mg/dL)**	-37.5 (114.4)	-47.4 (120.2)	0.4

*CKD indicates chronic kidney disease

Post-CHD event ACE inhibitor use was lower and calcium channel blocker use was higher in those with CKD versus those without CKD (Table 3). There were no statistically significant differences patients with and without CKD in post-CHD event exposure to statins, β-blockers, and ARBs (Table 3).

**Table 3:** Post-coronary heart disease event exposure of cardiovascular medications by CKD status.*

Medication Class	CKD (N = 159) Medication Possession Ratio (SD)	Non-CKD (N = 1088) Medication Possession Ratio (SD)	P Value
Statin	0.82 (0.25) N = 155	0.79 (0.27) N = 1034	0.3

β-blockers	0.78 (0.30) N = 146	0.74 (0.3) N = 1026	0.2

Calcium channel blockers	0.47 (0.38) N = 80	0.39 (0.37) N = 350	0.06

Angiotensin converting enzyme inhibitors	0.50 (0.39) N = 137	0.58 (0.38) N = 874	0.03

Angiotensin receptor blockers	0.50 (0.35) N = 36	0.52 (0.34) N = 203	0.8

*CKD indicates chronic kidney disease

The crude incidence of any hospitalized recurrent cardiovascular event (e.g., acute myocardial infarction, angina, coronary revascularization, peripheral arterial disease and stroke) and death were higher in CKD versus non-CKD patients (13.9 versus 11.5 per 100 person-years respectively, P < 0.001) (Table 4). In patients with CKD, there were a total of 307 total events (N = 23 acute myocardial infarction, N = 32 angina, N = 98 revascularization, N = 51 percutaneous coronary intervention, N = 53 coronary artery bypass surgery, N = 9 stroke, N = 5 peripheral vascular disease and N = 36 death) during follow-up. In patients without CKD, there were a total of 1,808 events (N = 86 acute myocardial infarction, N = 187 angina, N = 663 revascularization, N = 446 percutaneous coronary intervention, N = 260 coronary artery bypass surgery, N = 56 stroke, N = 17 peripheral vascular disease and N = 93 death) during follow-up.

**Table 4:** Incidence (per 100 person-years) of recurrent cardiovascular events and death by CKD status*

Cardiovascular Event	CKD (N = 159) Rate per 100 person years (95% Confidence Interval)	Non- CKD (N = 1088) Rate per 100 person years (95% Confidence Interval)
**Acute myocardial Infarction**	1.75 (1.10 to 2.62)	0.87 (0.70 to 1.08)

**Angina**	2.44 (1.67 to 3.45)	1.96 (1.69 to 2.26)

**Any revascularization**	10.15 (8.42 to 12.37)	9.58 (8.87 to 10.34)

**Percutaneous coronary intervention**	4.25 (3.17 to 5.60)	5.50 (5.01 to 6.04)

**Coronary artery bypass surgery**	4.48 (3.35 to 5.86)	2.89 (2.54 to 3.26)

**Stroke**	0.63 (0.29 to 1.21)	0.55 (0.42 to 0.71)

**Peripheral arterial disease**	0.35 (0.11 to 0.82)	0.16 (0.10 to 0.26)

**Death**	2.51 (1.76 to 3.47)	0.89 (0.73 to 1.10)

**Any Event**	13.89 (11.60 to 16.51)	11.47 (10.68 to 12.32)

*CKD indicates chronic kidney disease

Compared with eGFR ≥ 60 ml/min/1.73 m^2^, those in lower eGFR categories had higher relative rates of recurrent cardiovascular events or death (Table 5). After adjustment for patient characteristics (Model 2) and comorbid conditions (Model 3), the hazard ratio for recurrent cardiovascular event or death decreased for eGFR 45-59 ml/min/1.73 m^2 ^and eGFR < 45 ml/min/1.73 m^2^. Finally, after further adjustment for statin, β-blocker, calcium channel blocker, ACE inhibitor or ARB, there were no longer significant associations for eGFR 45-59 ml/min/1.73 m^2 ^or eGFR < 45 ml/min/1.73 m^2 ^(Table 5).

**Table 5:** Hazard ratio of recurrent cardiovascular event or death in ADVANCE cohort by eGFR (ml/min/1.73 m^2^)*

	Hazard ratio (95% CI) of recurrent cardiovascular event or death
**Model 1: Unadjusted Model**	

**eGFR > 60**	Referent

**eGFR 45-59**	1.61 (1.18 to 2.18)

**eGFR < 45**	1.85 (1.19 to 2.89)

**Model 2: Adjusted for age, sex, race/ethnicity, smoking, alcohol use, and physical activity**	

**eGFR > 60**	Referent

**eGFR 45-59**	1.52 (1.11 to 2.10)

**eGFR < 45**	1.70 (1.10 to 2.67)

**Model 3: Model 2 + adjusted for body mass index and history of diabetes and hypertension**	

**eGFR > 60**	Referent

**eGFR 45-59**	1.47 (1.10 to 2.02)

**eGFR < 45**	1.58 (1.00 to 2.50)

**Model 4: Model 3 + medication possession ratio of statins, β-blockers, calcium channel blockers, ACE inhibitors^†^/ARBs^‡^**	

**eGFR > 60**	Referent

**eGFR 45-59**	0.82 (0.25 to 2.66)

**eGFR < 45**	1.19 (0.25 to 5.58)

*eGFR indicates glomerular filtration rate

†ACE inhibitor indicates angiotensin converting enzyme inhibitor

‡ARB indicates angiotensin receptor blocker

## Discussion

Among a unique, well-characterized cohort of persons with incident CHD, we evaluated whether differential cardiovascular medication use may explain, at least in part, the excess risk of recurrent cardiovascular disease events and death in CKD patients with incident CHD. We found that in adults with newly recognized CHD, those with pre-existing CKD were less likely to receive post-event ACE inhibitors and more likely to receive calcium channel blockers. Blood pressure and lipid levels improved in those with CKD during the follow-up period suggesting that study participants were aggressively treated and doses were optimized in those who did receive treatment. Mild (eGFR 45-59 ml/min/1.73 m^2^) and moderate (eGFR < 45 ml/min/1.73 m^2^) CKD were strong predictors for subsequent cardiovascular events and death even after adjustment for patient characteristics, but this association was no longer significant and had estimates near the null after additional adjustment for post-event cardiovascular medication use. These results suggest that the excess risk of recurrent cardiovascular events and death in patients with CKD may be explained, in part, by the differential use of cardiovascular medications. To our knowledge, no previously published study has examined whether differential medication exposure can explain the excess burden of recurrent cardiovascular disease in CKD patients with known CHD.

In our study, the overall improvement in blood pressure and lipid levels in those with CKD from baseline to the end of follow-up suggests that those who were treated with cardiovascular medications were treated aggressively. However, we found that those with CKD were less likely to receive ACE inhibitors which may be a reflection of concern of drug-related adverse effects, such as hyperkalemia or a hemodynamically mediated decrease in eGFR. The non-significant difference between post-event ARB exposure in those with CKD versus those without CKD may be explained by the low overall use of ARBs in our sample. We observed no significant differences in the post-event receipt of statins and β-blockers between patients with or without pre-existing CKD which is consistent with aggressive secondary prevention efforts among all patients within this health care setting. The higher use of calcium channel blockers among those with CKD is consistent with the fact that it is often more difficult to control blood pressure among CKD patients.

Several studies have found that selected cardiovascular medications appear to be beneficial in patients with CKD. The Study of Heart and Renal Protection (SHARP) randomly assigned patients with advanced CKD or ESRD to ezetimibe plus simvastatin and found a reduction in LDL cholesterol levels as well as a reduction in cardiovascular events with this combination therapy compared with placebo [32]. Several observational studies as well have observed lower rates of cardiovascular outcomes in those with known CHD and CKD who received statin therapy [33-36]. In a post hoc analysis of the Heart Outcomes Prevention Evaluation (HOPE trial), ramipril was associated with a lower incidence of subsequent cardiovascular events in both mild CKD and non-CKD patients [37]. In the Survival And Ventricular Enlargement (SAVE) trial, post-myocardial randomization to captopril resulted in a reduction of cardiovascular events, regardless of renal function [38]. In contrast, a study of more than 6,000 heart failure patients with varying levels of renal function found that while users of ACE inhibitors without CKD had lower mortality compared with those who did not receive ACE inhibitor therapy, receipt of ACE inhibitors was not associated with mortality differences in the subset of patients with CKD (adjusted OR 1.21, 95% CI 0.97 to 1.51) [39]. However, the study participants had low ejection fraction, were followed for only 12 months and the study did not focus on incident ACE inhibitor therapy, raising the possibility of reverse causality or the presence of residual confounders (e.g. anemia, as hemoglobin data was not available). Studies have also found a survival benefit with β-blocker use in ESRD [40] and CKD [41]. The Bezafibrate Infarction Prevention (BIP) study found that in a cohort of 568 CKD patients with CHD, 43.1% used beta-blockers at baseline and use of beta-blockers was associated with a reduction of the rate of acute myocardial infarction or sudden cardiac death [41]. Our study is consistent with these results and extends our knowledge by also studying the influence of other commonly prescribed cardiovascular medications within a diverse, community-based sample of newly diagnosed CHD. While previous studies have examined the use of a single cardiovascular medication, we were able to study multiple cardiovascular medications that are commonly prescribed together.

Our study had several strengths. Our study population was a large and diverse sample of well-characterized patients. We had up to seven years of longitudinal data on medication use for the study population and were able to accurately calculate a medication possession ratio. Our study had a few limitations as well. Information on longitudinal use of aspirin was unavailable in our study population. We also could not completely account for potential confounding by indication for selected medications. For example, we could not control for clinical decisions such as withholding an ACE inhibitor in a patient with hyperkalemia. As with any observational study, we cannot prove the causal role of post-event medication use on recurrent cardiovascular outcomes, but we attempted to control for known major confounders and found that the unfavorable association between pre-existing CKD and outcomes was no longer significant after adjustment for post-event receipt of cardiovascular medications. Finally, we conducted our study among health plan members within a large integrated health care delivery system in northern California, so our findings may not be completely generalizable to other health care settings or to uninsured patients.

## Conclusions

In conclusion, our study suggests that differential cardiovascular medication use after an incident coronary heart disease event may explain, at least in part, the excess risk of recurrent cardiovascular events and death in patients with pre-existing chronic kidney disease. Although further randomized trials are needed to explore this association, our results suggest that patients with chronic kidney disease are at high risk for recurrent cardiovascular events and death and that more systematic use of selected cardiovascular medication may help to significantly attenuate this risk.

## Competing interests

The authors declare that they have no competing interests.

## Authors' contributions

NB was involved in conception and design of the study, data analysis, manuscript preparation and approved the final manuscript. CYH was involved in the conception and design of the study, data analysis, manuscript prepartion and approved the final manuscript. MC was involved in the acquisition of data, analysis of data, interpretation of data, manuscript preparation and approved the final manuscript. CI was involved in the data analysis, interpretation of data, manuscript preparation and approved the final manuscript. SP was involved in the data analysis, interpretation of data, manuscript preparation and approved the final manuscript. MH was involved in the data analysis, interpretation of data, manuscript preparation and approved the final manuscript. AG was involved in conception and design of the study, data acquisition, data analysis, manuscript preparation and approved the final manuscript

## Pre-publication history

The pre-publication history for this paper can be accessed here:

http://www.biomedcentral.com/1471-2369/12/44/prepub
